# Epidemiological trends and comparative forecasting models of human brucellosis in inner mongolia autonomous region, mainland China, 2004–2024

**DOI:** 10.1371/journal.pntd.0014439

**Published:** 2026-07-16

**Authors:** Na Zhang, Qiuju Yang, Chuizhao Xue, Zhiguo Liu, Na Ta, Zhenjun Li

**Affiliations:** 1 National Key Laboratory of Intelligent Tracking and Forecasting for Infectious Diseases, National Institute for Communicable Disease Control and Prevention, Chinese Center for Disease Control and Prevention & Chinese Academy of Preventive Medicine, Beijing, China; 2 School of Public Health, Inner Mongolia Medical University, Hohhot, People’s Republic of China; 3 Yunnan Provincial Key Laboratory for Natural Focal Disease Control and Prevention, Yunnan Institute of Endemic Disease Control and Prevention, Kunming, People’s Republic of China; 4 National Institute of Parasitic Diseases, Chinese Center for Disease Control and Prevention (Chinese Center for Tropical Diseases Research), Shanghai, China; 5 Inner Mongolia Autonomous Region Center for Disease Control and Prevention, South Section, Yongping Road (East Side), Xincheng District, Hohhot Inner Mongolia Autonomous Region China, People’s Republic of China; Colorado State University, UNITED STATES OF AMERICA

## Abstract

**Introduction:**

Brucellosis remains a severe zoonotic threat in the Inner Mongolia Autonomous Region of China.

**Methodology:**

This study integrates a comprehensive epidemiological trend analysis with a novel methodological comparison of forecasting techniques to inform control strategies.

**Results:**

Using reported human brucellosis surveillance data from Inner Mongolia for 2004–2024, joinpoint regression analysis revealed a persistently increasing yet fluctuating long-term trend (AAPC = 5.13%, *P* < 0.001), characterized by significant epidemic surges in 2004–2010 (APC = 22.43%, *P* < 0.001) and 2016–2021 (APC = 29.83%, *P* < 0.001), interrupted by a decline phase in 2010–2016 (APC = -17.17%, *P* < 0.001) and 2021–2024 (APC = -12.15). The disease demonstrated strong seasonality with June–August peaks, and predominance among farmers and herdsmen aged 30–60 years. Building on this epidemiological foundation, we rigorously compared the predictive performance of the standard Seasonal Autoregressive Integrated Moving Average (SARIMA) model against its Bootstrap-enhanced version for 24-month-ahead forecasting (2023–2024 validation). This finding offers a novel perspective on enhancing the predictive performance of brucellosis models. While the Bootstrap approach achieved superior point forecast accuracy by reducing Mean Absolute Error by 39.95% and Median Absolute Percentage Error by 33.55% compared to SARIMA, it produced severely overconfident prediction intervals, with only 33.33% empirical coverage compared to SARIMA’s 91.67%. This study validates the SARIMA model as a robust baseline for brucellosis forecasting and introduces a Bootstrap ensemble method as a powerful tool for significantly enhancing point prediction accuracy.

**Conclusion:**

The findings provide novel epidemiological insights that offer a scientific basis for disease control measures and decision-making. Future work should aim to develop hybrid models that bridge this gap, and delivering high accuracy in both point and interval forecasts.

## Author summary

Inner Mongolia Autonomous Region, a historical brucellosis hotspot and Chinese major livestock-producing region, has seen a sustained nonlinear rise in human brucellosis over two decades. Epidemic features include marked June–August seasonality, occupational clustering among herders and slaughterhouse workers, and concentration in rural adults aged 30–60 years. Our forecasting reveals a clear trade-off between predictive accuracy and interval reliability. The bootstrap-enhanced model reduced MAE by 39.95% and MdAPE by 47.96% relative to SARIMA yet yielded severely miscalibrated confidence intervals with only 33.33% empirical coverage, compared to 91.67% for SARIMA. We propose a practical dual-model strategy where the bootstrap-enhanced approach serves high-precision short-term forecasting for routine prevention and emergency management, while SARIMA is retained when credible uncertainty estimation is needed for risk communication, resource allocation, and evidence-based policy formulation. This framework balances rapid response and robust risk assessment for zoonotic disease control in pastoral settings.

## Introduction

Brucellosis is a globally distributed zoonosis that has a devastating impact on both human health and livestock economies, particularly in developing countries [1]. Brucellosis in European and North American countries have been under control, however, the high prevalence in some undeveloped regions of the world, such as Middle-East, Africa, and Asia, especially Syria, Iraq, Kazakhstan, and Chinese mainland [2,3]. The high incidence of human brucellosis continues to pose a significant threat to public health, imposing a substantial socioeconomic burden [4]. Inner Mongolia Autonomous Regions (Inner Mongolia) and bordering provinces/regions are high-risk areas for human brucellosis because animal husbandry is the major pillar industry and the main income source for most of the local population [5].

Inner Mongolia has long been a historical epicenter for both human and animal brucellosis. Following large-scale vaccination and surveillance programs in ruminants after the 1970s, the epidemic gradually declined, reaching its lowest point in 1980 with only three reported human cases [6]. However, a resurgence began around 2000, with cases rising sharply and peaking in 2010 at 19,141 reported infections [7]. During the period 2010–2015, the spatiotemporal distribution showed a clear seasonal pattern, with peaks occurring consistently between March and June, and cases concentrated predominantly among male farmers and herdsmen aged 40–59. Geographically, the Xilin Gol League and Hulunbuir City were identified as the primary high-risk areas [8].

In the subsequent five years (2016–2020), childhood brucellosis incidence in Inner Mongolia exhibited an upward trend and displayed spatial clustering [9]. Most recently, between 2019 and 2023, the disease continued to spread across northern China, with Inner Mongolia remaining the region bearing the highest reported case [10]. Long-term trend analyses and comparative forecasting studies for brucellosis in Inner Mongolia remain limited. This study addresses this gap by applying Joinpoint regression to identify key epidemic turning points, and systematically compares SARIMA and Bootstrap-enhanced forecasting methods to evaluate their performance trade-offs [11]. The findings provide evidence-based guidance for model selection in public health surveillance and early warning systems, supporting informed decision-making for brucellosis control.

## Methods

### Ethics statement

The study protocol was approved by the Ethics Committee of Inner Mongolia Autonomous Region Center for Disease Control and Prevention. All data were anonymous, no patients took part in this study, and informed consent to participate was not obtained.

### Data sources and process

The number of reported cases and incidence rate of human brucellosis from 2004 to 2024 were extracted from Chinese National Notifiable Disease Reporting System (NNDRS), established in 2004. The sex- and age-specific case data have been collected since 2005. The population statistics and livestock population (e.g., sheep, goats and cattle stocks) were sourced from the Inner Mongolia Statistical Yearbook. Excel 2021 software (Microsoft, Redmond, WA, USA) and R software (version 4.4.1; Bell Laboratories, New Jersey, America) were used for data processing. At a significance level of 0.05, the chi-square test was performed using R to compare incidence rates across gender and age groups. Pearson correlation analysis was performed to examine the relationship between human brucellosis incidence and livestock population (e. g., sheep, goats and cattle stocks) in Inner Mongolia. A correlation coefficient r ≥ 0.8 was considered a remarkably strong correlation. The forecast package in R was employed to decompose the month time series data using the Seasonal-Trend decomposition procedure based on Loess (STL). STL is a time series decomposition method that separates a time series into three key components: trend, seasonal, and remainder. Inner Mongolia’s twelve prefecture-level cities were grouped into two regions: Eastern (Hulun Buir, Hinggan League, Tongliao, Chifeng, Xilingol League) and Western (Alxa League, Wuhai, Ordos, Bayan Nur, Baotou, Hohhot, Ulanqab).

### Joinpoint regression analysis

The incidence trend analysis was conducted using Joinpoint Regression Program [12] (version 5.2.0; Information Management Services, Inc., Calverton, MD, USA), developed by the Statistical Research and Applications Branch of the Surveillance Research Program at the U.S. National Cancer Institute. Briefly, the annual percentage change (APC) and the average annual percentage change (AAPC) for each segment were estimated using Joinpoint regression, which focused on estimating the temporal trends in the incidence rate of human brucellosis. WBIC is recommended because it achieves computational efficiency far superior to the permutation test while maintaining a similar probability of correct selection and controlling overfitting, and it offers the greatest flexibility across different situations [13]. The maximum number of join points was set to three, in accordance with the software’s algorithmic recommendations that scale this parameter with the number of data points [14].

### Construction of the SARIMA Model of human brucellosis from 2004 to 2024

All statistical analyses were performed using R software (version 4.4.1), utilizing the tseries, forecast, and Metrics packages. The study analyzed the monthly incidence rates of human brucellosis in the Inner Mongolia Autonomous Region from 2004 to 2024. A Seasonal Autoregressive Integrated Moving Average (SARIMA) model was constructed to fit the time series data. Because the time series decomposition and statistical description of incidence data mentioned earlier have confirmed that brucellosis has a clear seasonality. The model structure is denoted as SARIMA (p, d, q) (P, D, Q) s, where (p, d, q) represents the non-seasonal parameters, (P, D, Q) represents the seasonal parameters, and s denotes the seasonal period (set to s = 12 for monthly data). The model was used to predict the incidence of brucellosis for the period 2025–2027.

### Data preparation and checking

The dataset was partitioned into two segments: data from January 2004 to December 2022 (228 months) served as the training set for model construction and parameter estimation; data from January 2023 to December 2024 (24 months) served as the validation set to evaluate out-of-sample predictive performance. Stationarity and White Noise Testing: The Augmented Dickey-Fuller (ADF) test was applied to the training set to assess stationarity [15]. Non-stationary sequences underwent differencing (including seasonal differencing) to eliminate trend and seasonal effects. Subsequently, the Ljung-Box test (Q statistic) was employed to verify that the sequence was not pure white noise, thereby confirming the presence of extractable information [16].

### Bootstrap-enhanced SARIMA model

Brucellosis data may exhibit sudden changes due to policy interventions or under certain circumstances, in which case traditional models based on the assumption of stationarity would become invalid. The bootstrap method, however, can enhance the randomness of sample parameters by generating a large number of possible scenarios. By randomly sampling parameters from a parameter pool to adjust the model, this approach can better characterize the uncertainty associated with potential changes in future patterns, thereby yielding results that are more consistent with real-world conditions. Therefore, to quantify prediction uncertainty and enhance stability, a bootstrap resampling technique was implemented. We hypothesize that the SARIMA-Bootstrap hybrid model will outperform the single SARIMA benchmark model in terms of model evaluation metrics and will enhance model predictive performance.

The introduction of the bootstrap method serves as an enhancement to the SARIMA model, resulting in a combined bootstrap-enhanced SARIMA. This approach generates 1, 000 random samples with replacement based on the fitted residuals from the optimal original model. The resampled residual sequences are then added back to the fitted values of the original model, thereby creating 1, 000 new bootstrap time series datasets [17]. Confidence intervals and hypothesis testing typically require a relatively large sample size for resampling, often at least 1,000 [18]. Comparing model evaluation metrics between sample sizes of 1,000 and 10,000 reveals no significant changes. For each dataset, parameters were refitted while maintaining the original model structure, followed by point forecasts for the subsequent validation and prediction data. The median of these 1,000 forecasts was selected as the final ensemble prediction value. Additionally, the 2.5th and 97.5th percentiles were calculated to construct the 95% Confidence Interval (95% CI). The bootstrap confidence intervals are constructed from percentiles of residual-resampled forecasts, inheriting all structural SARIMA assumptions.

### Model identification and parameter estimation

Automatic model identification and selection were performed using the auto.arima function from the forecast package. Based on the Hyndman-Khandakar algorithm, this function searches over possible (p,d,q) (P,D,Q)s parameter combinations [19]. Model selection was guided by the corrected Akaike Information Criterion (AICc), which offers greater robustness than AIC in small-sample contexts [20]. The modeling process incorporated the seasonal periodicity of the data (period = 12) and automatically determined the optimal differencing orders (d and D) as well as the autoregressive and moving average orders.

### Model diagnostics

Residual diagnostics were performed using the check residual’s function. This included the analysis of residual time plots, autocorrelation function (ACF) and histogram of residuals, and the Ljung-Box test to verify that the residuals satisfied the white noise assumption.

### Model evaluation metrics

Predictive accuracy was evaluated using the validation set, focusing on the Mean Absolute Error (MAE) and the Median Absolute Percentage Error (MdAPE). Following public health forecasting standards, MdAPE is used to measure the median level of forecast errors and possesses a strong ability to resist outliers. However, it only reflects the central position of the error distribution and is insensitive to extreme errors. Therefore, when evaluating models, MdAPE should be considered in conjunction with other metrics such as MAE and MAPE for comprehensive judgment [21]. Since no unified evaluation standard for MdAPE has yet been established in the existing literature—where smaller values are generally considered better—this study refers to the empirical classification of MAPE (Lewis, 1982). Accordingly, an MdAPE of less than 10% is regarded as highly accurate, 10%–20% as good, 20%–50% as reasonable, and greater than 50% as indicating poor forecasting performance [22]. Furthermore, the improvement in uncertainty quantification was assessed by comparing error metrics between the bootstrap ensemble prediction and the single model prediction.

## Results

### Epidemic trends of human brucellosis in Inner Mongolia

**Fig 1:**
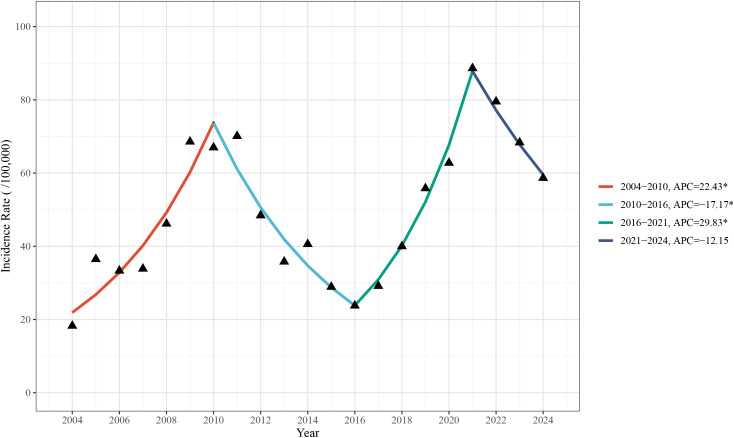
Joinpoint regression analysis of human brucellosis incidence in Inner Mongolia Autonomous Region, China, 2004–2024. Note: APC, annual percent change. *: Statistically Significant at alpha = 0.05 (*P* < 0.05).

From 2004 to 2024, the incidence of human brucellosis exhibited a significantly fluctuating upward trend overall (AAPC = 5.13*, *P* < 0.001), with signs of a resurgence emerging in recent years (Table 1). It revealed significant increases in 2004–2010 (APC = 22.43*, *P* < 0.001) and 2016–2021 (APC = 29.83*, *P* < 0.001), but a decline in 2010–2016 (APC = -17.17*, *P* < 0.001) and 2021–2024 (APC = -12.15) (Fig 1). The annual incidence rate increased from 23.00/100,000 (n = 4,423) in 2004 to 58.65/100,000 (n = 14,053) in 2024. This re-emerging epidemic trend was consistent with the increase in sheep and goat populations over the past two decades (S1A Fig). Pearson correlation analysis showed that the stocks of sheep in Inner Mongolia were strongly positively correlated with the human incidence rate (S1B Fig).

**Table 1:** Join point regression analysis of human brucellosis in Inner Mongolia Autonomous Region from 2004–2024.

	Model	Segment	Segment Start	Segment End	Value	95% LCI	95% HCI	*P*
APC								
	3	0	2004	2010	22.43*	15.35	31.30	0.002
	3	1	2010	2016	-17.17*	-25.34	-11.87	0.002
	3	2	2016	2021	29.83*	20.52	49.61	0.002
	3	3	2021	2024	-12.15	-29.33	0.39	0.061
AAPC								
	3	0	2004	2024	5.13*	3.53	6.71	0.000

Note: *: Statistically Significant; LCI, Low confidence interval; HCI, High confidence interval; APC, Annual percent change; AAPC, Average annual percent change.

### Gender, age, occupation and season distribution profile of human brucellosis

**Fig 2:**
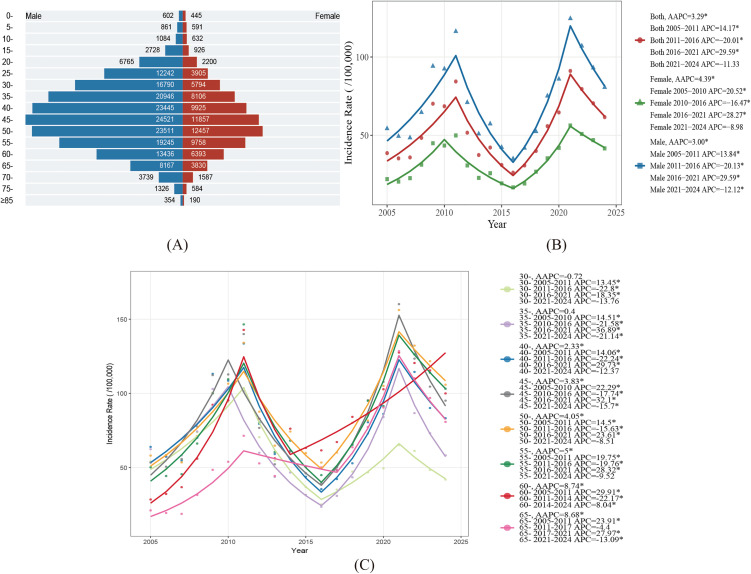
(A) Pyramid distribution of brucellosis cases by gender and age group in Inner Mongolia, 2005–2024; (B) Joinpoint trend chart of brucellosis incidence by gender in Inner Mongolia, 2005–2024; (C) Joinpoint trend chart of brucellosis incidence in the eight age groups in Inner Mongolia, 2005–2024; Note: APC, annual percent change. AAPC, average annual percent change. *: Statistically Significant at alpha = 0.05 (*P* < 0.05).

**Fig 3:**
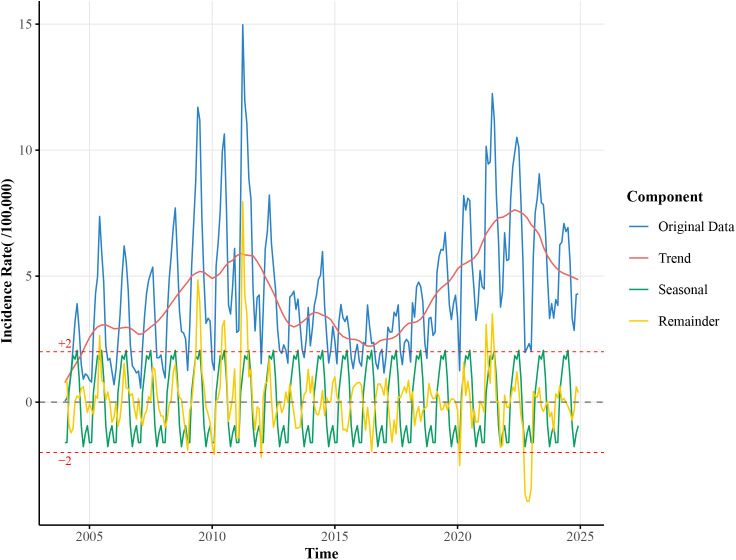
STL time series decomposition of brucellosis incidence in Inner Mongolia, 2004–2024. Note: STL, seasonal and trend decomposition using Loess. The two red dashed lines represent the upper and lower control limits of the residual component. Points outside these limits indicate anomalous fluctuations not explained by the seasonal and trend components. All components of the time series decomposition share the same y‑axis scale.

Over 2005–2024, 259,104 total cases were documented; 179,869 were male (69.42%) and 79,235 female (30.58%), with a male-to-female ratio of 2.27:1 (χ² = 33730.67, *P* < 0.001) (Fig 2A). Temporal epidemic patterns were highly similar between sexes, with marked increases during 2005–2011 and 2016–2021 and subsequent declines in 2011–2016 and 2021–2024 (Fig 2B). Cases covered all age groups from 0 to ≥ 85 years (χ² = 88405, *P* < 0.001), with case counts per group ranging from 38 to 36,378. The 30–60 years age group contributed 79.58% of all cases (206,184/259,104). The 45 years group had the largest case number, followed by the 50- and 40-years groups (35,968 and 33,370, respectively) (Fig 2A). Joinpoint regression across 18 age groups showed consistent temporal trends in 14 groups (S2 Fig). The 60 years group presented a distinct trajectory among high-incidence age groups, with two significant rising phases during 2005–2011 and 2016–2024 (Fig 2C). Regarding occupational distribution, farmers constituted the majority of cases (71.82%), followed by herdsmen (16.05%) and students (1.47%); all other 15 occupations individually accounted for below 5% of cases (S3 Fig). The STL decomposition of monthly incidence rates showed a rising trend during 2004–2010, fluctuating levels in 2011–2015, and a sustained overall increase from 2016 to 2024 with a prominent peak in 2020–2022. Obvious annual seasonality was detected, with incidence peaks concentrated in summer (June–August) and troughs in winter (December–February). Residual analysis confirmed the model effectively captured trend and seasonality, with residuals behaving mostly as random fluctuations. Two prominent outliers were detected, a large positive residual in April 2011 and a marked negative residual in February 2020 (Fig 3).

### Joinpoint regression analysis of human brucellosis in 11 cities from 2004 to 2024

**Fig 4:**
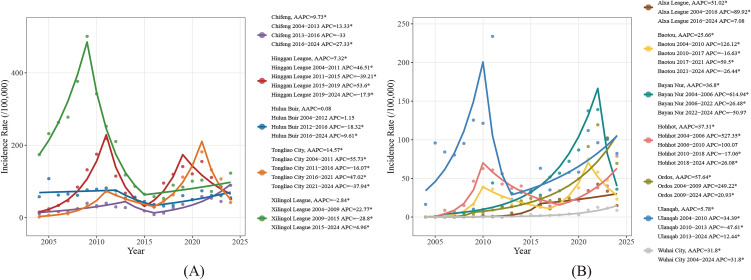
The joinpoint regression analysis of human brucellosis in eastern (A) and western (B) in Inner Mongolia from 2004 to 2024. Note: APC, the annual percentage change; AAPC, the average annual percentage change. *: Statistically Significant at alpha = 0.05 (P < 0.05).

Joinpoint regression identified marked disparities in human brucellosis epidemic trends between eastern and western Inner Mongolia. Three join points were detected in Hinggan League and Tongliao City within eastern regions, with incidence presenting fluctuating rising and falling patterns (Fig 4A, Table 2). Most eastern regions showed a steady increasing incidence trend with AAPC values ranging from 0.08 to 14.57, while only Xilingol League experienced a declining trend (AAPC = -2.84, Table 3). By contrast, all western regions exhibited significant upward trends in incidence, with AAPC values of 5.78 to 57.64 (all *P* < 0.01) (Table 3).

**Table 2:** Annual percentage change (APC) of human brucellosis in 12 cities (leagues) in Inner Mongolia Autonomous Region from 2004–2024.

Area	Cohort	Segment	Period	APC	95%CI	*P*
Western	Alxa League - 1 Joinpoint	1	2004-2016	89.92*	55.99 ~ 1810.50	0.004
		2	2016-2024	7.08	-91.70 ~ 53.01	0.802
	Baotou - 3 Joinpoints	1	2004-2010	126.12*	98.32 ~ 154.00	< 0.001
		2	2010-2017	-16.63*	-30.99 ~ -7.65	0.006
		3	2017-2021	59.50*	29.78 ~ 108.54	0.006
		4	2021-2024	-26.44*	-49.48 ~ -7.23	0.012
	Bayan Nur - 2 Joinpoints	1	2004-2006	614.94*	238.96 ~ 974.47	< 0.001
		2	2006-2022	26.48*	21.07 ~ 35.49	0.005
		3	2022-2024	-50.97	-66.55 ~ 4.47	0.065
	Hohhot - 3 Joinpoints	1	2004-2006	527.35*	210.96 ~ 1129.99	< 0.001
		2	2006-2010	100.07	-17.00 ~ 155.37	0.086
		3	2010-2018	-17.06*	-46.34 ~ -1.55	0.046
		4	2018-2024	26.08*	4.14 ~ 117.34	0.032
	Ordos - 1 Joinpoint	1	2004-2009	249.22*	142.49 ~ 665.91	< 0.001
		2	2009-2024	20.93*	10.24 ~ 30.84	0.002
	Ulanqab - 2 Joinpoints	1	2004-2010	34.39*	16.23 ~ 90.10	0.003
		2	2010-2013	-47.61*	-58.40 ~ -15.63	0.003
		3	2013-2024	12.44*	4.93 ~ 30.03	0.004
	Wuhai City - 0 Joinpoints	1	2004-2024	31.80*	16.22 ~ 48.80	< 0.001
Eastern	Chifeng - 2 Joinpoints	1	2004-2013	13.33*	1.52 ~ 79.66	0.038
		2	2013-2016	-33.00	-48.33 ~ 9.24	0.062
		3	2016-2024	27.33*	5.52 ~ 116.44	0.038
	Hinggan League - 3 Joinpoints	1	2004-2011	46.51*	34.04 ~ 62.40	0.001
		2	2011-2015	-39.21*	-55.51 ~ -24.98	0.002
		3	2015-2019	53.60*	27.74 ~ 112.15	0.002
		4	2019-2024	-17.90*	-33.88 ~ -8.04	0.003
	Hulun Buir - 2 Joinpoints	1	2004-2012	1.15	-4.96 ~ 25.89	0.486
		2	2012-2016	-18.32*	-31.36 ~ -3.15	0.042
		3	2016-2024	9.61*	2.02 ~ 34.08	0.035
	Tongliao City - 3 Joinpoints	1	2004-2011	55.73*	40.60 ~ 82.34	0.002
		2	2011-2016	-16.07*	-40.67 ~ -0.31	0.046
		3	2016-2021	47.02*	25.76 ~ 109.16	0.002
		4	2021-2024	-37.94*	-62.68 ~ -15.52	0.003
	Xilingol League - 2 Joinpoints	1	2004-2009	22.77*	10.69 ~ 40.13	< 0.001
		2	2009-2015	-28.80*	-39.47 ~ -22.23	< 0.001
		3	2015-2024	4.96*	0.04 ~ 11.47	0.048

Note: APC, Annual percentage change; *: Statistically Significant at alpha = 0.05 (*P* < 0.05); CI, confidence interval.

**Table 3:** Average annual percent change (AAPC) of human brucellosis in 12 cities (leagues) of Inner Mongolia Autonomous Region from 2004 to 2024.

	Cohort	Period	AAPC	95%CI	*P*
Western	Alxa League - 1 Joinpoint	2004-2024	51.02*	20.62 ~ 94.13	< 0.001
	Baotou - 3 Joinpoints	2004-2024	25.66*	21.55 ~ 29.26	< 0.001
	Bayan Nur - 2 Joinpoints	2004-2024	36.80*	29.34 ~ 44.87	< 0.001
	Hohhot - 3 Joinpoints	2004-2024	37.31*	30.58 ~ 45.93	< 0.001
	Ordos - 1 Joinpoint	2004-2024	57.64*	47.45 ~ 68.37	< 0.001
	Ulanqab - 2 Joinpoints	2004-2024	5.78*	2.25 ~ 10.82	0.004
	Wuhai City - 0 Joinpoints	2004-2024	31.80*	16.22 ~ 48.80	< 0.001
Eastern	Chifeng - 2 Joinpoints	2004-2024	9.73*	4.49 ~ 16.83	0.020
	Hinggan League - 3 Joinpoints	2004-2024	7.32*	3.84 ~ 10.17	< 0.001
	Hulun Buir - 2 Joinpoints	2004-2024	0.08	-1.92 ~ 2.92	0.699
	Tongliao City - 3 Joinpoints	2004-2024	14.57*	10.34 ~ 18.85	< 0.001
	Xilingol League - 2 Joinpoints	2004-2024	-2.84*	-4.57 ~ -0.78	0.006

Note: AAPC, Average annual percent change; *: Statistically Significant at alpha = 0.05 (*p* < 0.05). CI, confidence interval.

The three join point trends were recorded in Baotou City, where the epidemic disease exhibited a fluctuating pattern of repeated rises and declines (Fig 4B and Table 2). In Bayan Nur, the disease incidence showed a persistent increase from 2004 to 2022 (APC [2004–2006] = 614.94*, *P* < 0.001; APC [2006–2022] = 26.48*, *P* < 0.01), followed by a decline from 2022 to 2024. Ulanqab demonstrated a fluctuating upward trend (APC [2004–2010] = 34.39*, *P* < 0.01; APC [2013–2024] = 12.44*, *P* < 0.01), while significant increases in incidence were observed in Wuhai City (APC = 31.80*, *P* < 0.001), Ordos (APC [2004–2009] = 249.22*, *P* < 0.001; APC [2009–2024] = 20.93*, *P* < 0.001), and Alxa League (APC [2004–2016] = 89.92*, *P* < 0.01) (Fig 4B).

### Stationarity testing for epidemic time-series properties

**Fig 5:**
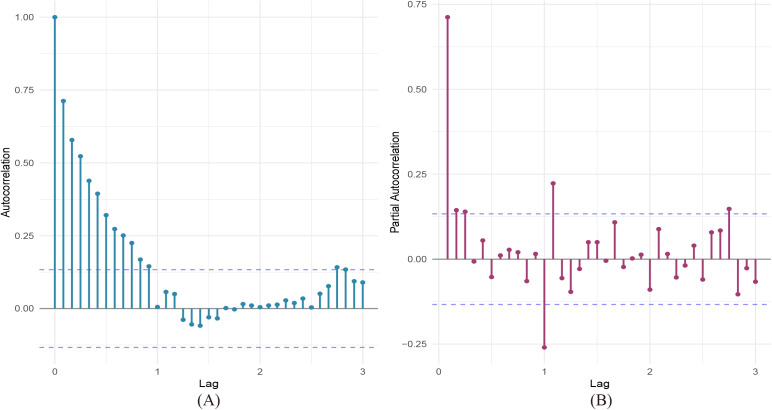
Autocorrelation (A) and partial autocorrelation (B) functions of seasonal first differences human brucellosis incidence. Note: The blue lines represent the 95% confidence bounds. The residuals are largely consistent with white noise, indicating that there is no significant autocorrelation in the residual series and that the model has passed the white noise test.

The training and validation sets maintained similar time-series characteristics before and after the split point (December 2022). The ADF test yielded a *p*-value of 0.0535, which lies at the borderline of statistical significance. This suggests that the series can be considered non-stationary, though the conclusion carries some uncertainty. Given this marginal result, the KPSS test (with the null hypothesis of stationarity, *P*  = 0.0232) was also employed as a cautious check to ensure robustness in subsequent modeling. The Ljung-Box test yielded a *p* < 0.05, confirming the series was not white noise and was suitable for modeling. After first-order seasonal differencing, the ACF showed significance at lag 1 (r = 0.50) followed by rapid decay (Fig 5A), while the PACF showed significance at lag 1 (ϕ = 0.50) followed by a cut-off (Fig 5B). No significant seasonal autocorrelation was observed in the lags. These features suggested a short-term memory effect, indicating the suitability of an ARIMA (p, 0, q) × (0, 1, Q) _12_ type model.

### Fitting performance of the SARIMA model

**Fig 6:**
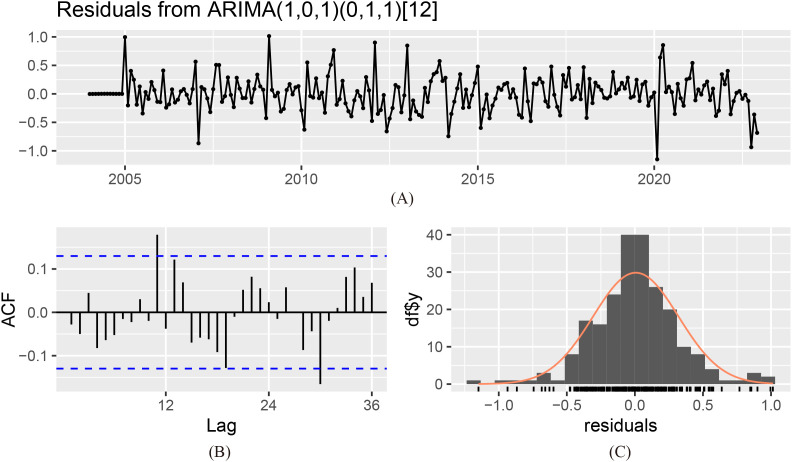
Diagnostic plots: ARIMA (1,0,1) (0,1,1) ₁₂ model residuals. **(A)** Time series plot of residuals. **(B)** ACF of residuals. **(C)** Histogram of residuals (with normal curve). Note: The blue lines represent the 95% confidence bounds in **B.** The residuals are largely consistent with white noise, indicating that there is no significant autocorrelation in the residual series and that the model has passed the white noise test.

The auto. arima algorithm selected ARIMA (1,0,1) (0,1,1) ₁₂ as the optimal model, with Box‑Cox transformation (λ = 0.27) applied for variance stabilization (Fig 6A). All model parameters were statistically significant (*P* < 0.001): non‑seasonal AR (1) = 0.97 (SE = 0.024), indicating strong positive correlation with monthly lagged incidence; non‑seasonal MA (1) = −0.39 (SE = 0.08), reflecting short‑term random shock correction; seasonal SMA (1) = −0.65 (SE = 0.07), suggesting prominent annual seasonal autocorrelation. Model fitness was supported by AIC = 138.87, AICc = 139.06 and BIC = 152.37. The Ljung–Box test for residuals presented *P* = 0.08 (> 0.05) (Fig 6B), and the residual histogram showed an approximately normal distribution (Fig 6C), verifying white noise residuals and adequate information extraction. Model projections indicated Inner Mongolia’s 2025 brucellosis incidence will follow typical seasonality, with higher transmission from March to August and the peak rate of 9.37 per 100,000 (95% CI: 1.31–16.70) expected in July.

### Prediction performance and bootstrap optimization

**Fig 7:**
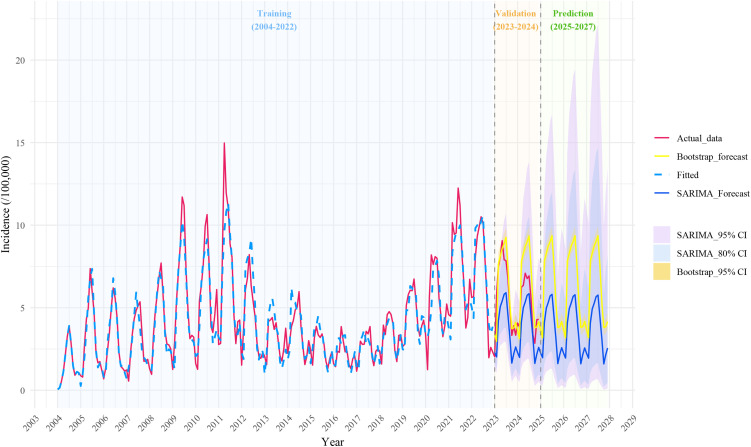
Model performance of SARIMA and Bootstrap-Enhanced SARIMA for brucellosis incidence forecasting on validation and prediction. Note: different background colors are used to indicate the data segments: blue represents the training set (2004–2022), orange represents the validation set (2023–2024), and green represents the forecast set (2025–2027).

The single SARIMA model produced a Mean Absolute Error (MAE) of 1.80 per 100,000 population, a Mean Absolute Percentage Error (MAPE) of 33.09%, and a Median Absolute Percentage Error (MdAPE) of 34.62% on the 2023–2024 validation set, falling within the reasonable accuracy range. The introduction of bootstrap resampling significantly enhanced predictive performance: MAE decreased to 1.08 per 100,000 (substantially lower than the single SARIMA model). MAPE decreased to 21.99%, representing a relative reduction of 33.55%. MdAPE decreased to 18.02%, representing a relative reduction of 47.96% and elevating the prediction accuracy（S1Table). While both modeling approaches project similar temporal trends and seasonal patterns for 2025–2027, the Bootstrap method consistently predicts higher incidence rates with greater precision (Fig 7).

## Discussion

In this study, using human brucellosis surveillance data from Inner Mongolia from 2004 to 2024, joinpoint regression revealed a persistently increasing yet fluctuating long-term trend. Before the 1980s, epidemic of human and animal brucellosis was severe in Inner Mongolia, China [23]. During the 1980s, the incidence of human and animal brucellosis was relatively low and seemed to decrease during the decade [6]. Since 1995, the incidence of human brucellosis has increased and is a re-emerging epidemic in Inner Mongolia due to animal husbandry boosting. The prevalence of human brucellosis in Inner Mongolia has increased during the past two decades, and high-high clusters areas are mainly concentrated in counties with extensive prairies and livestock [24]. This increasing trend is closely associated with multiple socioeconomic factors, including expanded sheep production, which in turn increases brucellosis transmission risks due to larger animal reservoirs and heightened human-livestock contact [25]. For instance, the population of sheep and goats in Inner Mongolia rose from 51.234 million in 2004 to 61.382 million in 2021. The expansion of small ruminant farming has facilitated the maintenance and widespread transmission of *Brucella* among both animal and human population. Therefore, successful brucellosis mitigation strategies necessitate institutionalized cooperation between veterinary services, human healthcare systems, and livestock production regulators, as fragmented approaches have proven ineffective in endemic settings [26].

Human brucellosis exhibits a significant seasonal, occupational, and age-related patterns. In Hulun Buir City, most reported cases were observed from April to June, indicating that these cases were closely related to the lambing season and shearing of domestic livestock [27]. The November epidemic peak closely correlates with local livestock production cycles. The annual slaughter season increases human exposure to infected animals via high‑risk activities such as slaughtering, lambing and veterinary work, while inadequate protection during winter meat and dairy processing further elevates transmission risks. A marked drop in brucellosis incidence occurred in February 2020 followed by a rapid resurgence from February 2021 onward. This temporal pattern aligns with established COVID-19-related disruptions to routine surveillance and medical attendance, which interfered with disease transmission dynamics [28]. A sharp 2021 brucellosis resurgence was mainly driven by relaxed mobility and social distancing controls, revived livestock trading, and resumed routine surveillance. The subsequent declining trend was linked to a range of prevention and control efforts including livestock vaccination and intensified population-oriented livestock tracking and intervention.

In Qinghai, age of the cases ranged from 8 years to 82 years, and the male to female ratio of the cases was 1.8∶1 (374/203), the prevalence rate in herdsman (47.83%, 276/577) was highest among different occupational populations [29]. In Xinjiang, human brucellosis was mainly concentrated in the 35–60 age group, accounting for 70.91% (412 cases), and the occupational distribution was mainly farmers, accounting for 43.20% [30]. In this study, 60- age group exhibiting a unique change trajectory, a survey shows that the utilization rate and standardized utilization rate of personal protective equipment (PPE) were lower in people over 60 years old, farmers, and those with lower educational level [31]. Furthermore, this elevated risk may also be associated with the general decline in physical condition and reduced immune function that typically accompanies aging in this population [32]. These results underscore the urgent requirement for targeted personal protective equipment (PPE) distribution and comprehensive training on proper PPE utilization among high-risk occupational populations during brucellosis epidemic phases, which could significantly reduce disease spread.

This study successfully constructed a SARIMA model to capture the distinct seasonal and trend patterns of human brucellosis incidence in Inner Mongolia. The optimal ARIMA model, enhanced with a Box-Cox transformation, provided an acceptable predictive performance, a finding that aligns with a substantial body of literature confirming the utility of SARIMA models for brucellosis forecasting in Chinese mainland [33]. The model’s parameters effectively captured the strong monthly autocorrelation and significant annual seasonality characteristic of the disease’s transmission dynamics, which are closely linked to livestock breeding cycles and human occupational exposure [34]. SARIMA models marginally improve HB forecasting accuracy over SARIMA by addressing long-range dependence in Henan’s seasonally patterned and rapidly increasing (34.9% annually post-2018) HB incidence data, though prediction reliability remains limited, necessitating hybrid models with environmental/livestock data and urgent public health interventions including livestock vaccination, education on unpasteurized dairy risks, and enhanced surveillance [35]. We note that projections beyond the validation period (i.e., 2025–2027) should be interpreted cautiously. This is because potential structural changes in livestock production, adjustments to surveillance practices, or shifts in public health policies may inadvertently affect the reliability of our long-term forecasts.

The primary contribution of our research is the novel application and evaluation of a bootstrap resampling technique to enhance the predictive accuracy of the traditional SARIMA framework. Our results clearly demonstrate that the bootstrap ensemble method yielded a marked improvement in point prediction accuracy, reducing the MAE by 40.0% and the MdAPE by 47.9% compared to the single SARIMA model. Although self-service resampling achieves a good fit in point estimation, the 95% confidence intervals in the validation set show considerable deviation from the actual values. This discrepancy arises because the confidence intervals are derived from the 95th percentile of 1,000 resampling iterations, and our data are relatively stable, with the SARIMA model assuming linear relationships. The bootstrap-enhanced SARIMA model achieved merely 33.3% empirical coverage for the nominal 95% confidence interval, revealing severe interval miscalibration. Key contributing factors include residual structure, heteroscedasticity and transformation bias, while conventional residual resampling has obvious limitations. Approaches such as block bootstrap, bias correction and hybrid models are viable optimizations that require further validation.

In a comparative time-series analysis of human brucellosis in the Ili Kazakh Autonomous Prefecture of Xinjiang, the XGBoost model demonstrated superior performance for long-term forecasting, achieving a coefficient of determination (R²) of 0.8033 and significantly outperforming the SARIMA (R² = 0.62) and LSTM (R² = 0.652) models, despite all three approaches exhibiting consistently low error metrics such as MAE, RMSE, and SMAPE [36]. Epidemiological and time series analysis of human brucellosis in Tebessa province, Algeria (2000–2020) demonstrated that both SARIMA and hybrid SARIMA-NNAR models enable highly accurate predictions of cases, which when combined with incidence mapping, provide valuable support for veterinary and health policymakers to develop informed, effective, targeted policies and timely interventions [37]. Our analysis indicates that while both the SARIMA and Bootstrap models capture similar epidemic patterns for 2025–2027, the Bootstrap method provides more precise projections with consistently higher incidence estimates. The SARIMA model is appropriate for brucellosis prediction, and the bootstrap resampling method offers a favorable improvement in predictive performance, advancing research on brucellosis modeling and performance enhancement. Nevertheless, the confidence intervals generated by the bootstrap resampling method are misleadingly narrow and should be used with caution. Furthermore, this method lacks generalizability at present and necessitates further validation supported by additional data.

Although this study yields meaningful findings, several limitations remain to be acknowledged. The analysis relied exclusively on historical incidence data and did not incorporate key contextual factors such as climatic conditions and livestock scale. Additionally, despite promising fitting performance, the generalizability of the bootstrap-enhanced SARIMA model requires further validation using multisource datasets and alternative modeling approaches. Future research should integrate covariates including temperature, precipitation and livestock inventory, adopt nonlinear models such as LSTM and Prophet, and incorporate geographic information to develop advanced spatiotemporal joint prediction frameworks.

## Conclusion

Human brucellosis in Inner Mongolia exhibits a complex, fluctuating long-term increase (AAPC = 5.13%, 2004–2024) with distinct seasonal, occupational, and demographic concentration. Forecasting evaluations reveal a critical methodological trade-off: a Bootstrap resampling approach significantly outperforms standard SARIMA in point forecast accuracy (reducing MAE by 39.95%, MAPE by 33.55% and MdAPE by 47.96%), but its prediction intervals are severely overconfident (33.3% empirical coverage vs. SARIMA’s 91.7%). The SARIMA time series model effectively fits and predicts brucellosis incidence data in Inner Mongolia, while the SARIMA-Bootstrap model exhibits enhanced forecasting stability. This contributes to the body of research on brucellosis prediction models and offers new perspectives for methodological advancements in predictive modeling.

## Supporting information

S1 Fig Temporal trends of brucellosis incidence and livestock population (A), and their correlation analysis (B) in the study area from 2000 to 2023. Note: (A) Temporal trends of brucellosis incidence rate (red line, per 100,000 population) and livestock population (right y-axis, in 10,000 heads) including sheep and goats (gray), cattle (purple), sheep (orange), goats (green), and hogs (blue), 2000–2023. (B) Correlation matrix showing Pearson’s correlation coefficients between brucellosis incidence and livestock populations. Blue/red colors denote positive/negative correlations, with intensity proportional to correlation magnitude. Significance levels: *p < 0.05, **p < 0.01, ***p < 0.001.
(TIF)

S2 Fig (Left) Joinpoint trend chart of brucellosis incidence in the age group (<45 years) in Inner Mongolia, 2005–2024; (Right) Joinpoint trend chart of brucellosis incidence in the age group (≥45 years) in Inner Mongolia, 2005–2024. Note: APC, annual percent change. AAPC, average annual percent change. *: Statistically Significant at alpha = 0.05 (*P* < 0.05).
(TIF)

S3 Fig Temporal clustering heatmap of human brucellosis cases by occupation, 2004–2024. Note: Heatmap rows represent years, columns represent occupations. The color scale indicates relative case proportions (red = high, blue = low).
(TIF)

S1 Table Comparison of prediction performance across four key metrics for the SARIMA and Bootstrap models. (XLSX)
