# Supplementary figures and images for: Epidemiological trends and comparative forecasting models of human brucellosis in inner mongolia autonomous region, mainland China, 2004–2024
Source: PLoS Negl Trop Dis. 2026 Jul 16;20(7):e0014439. doi: 10.1371/journal.pntd.0014439 (PMC13375022; doi:10.1371/journal.pntd.0014439)

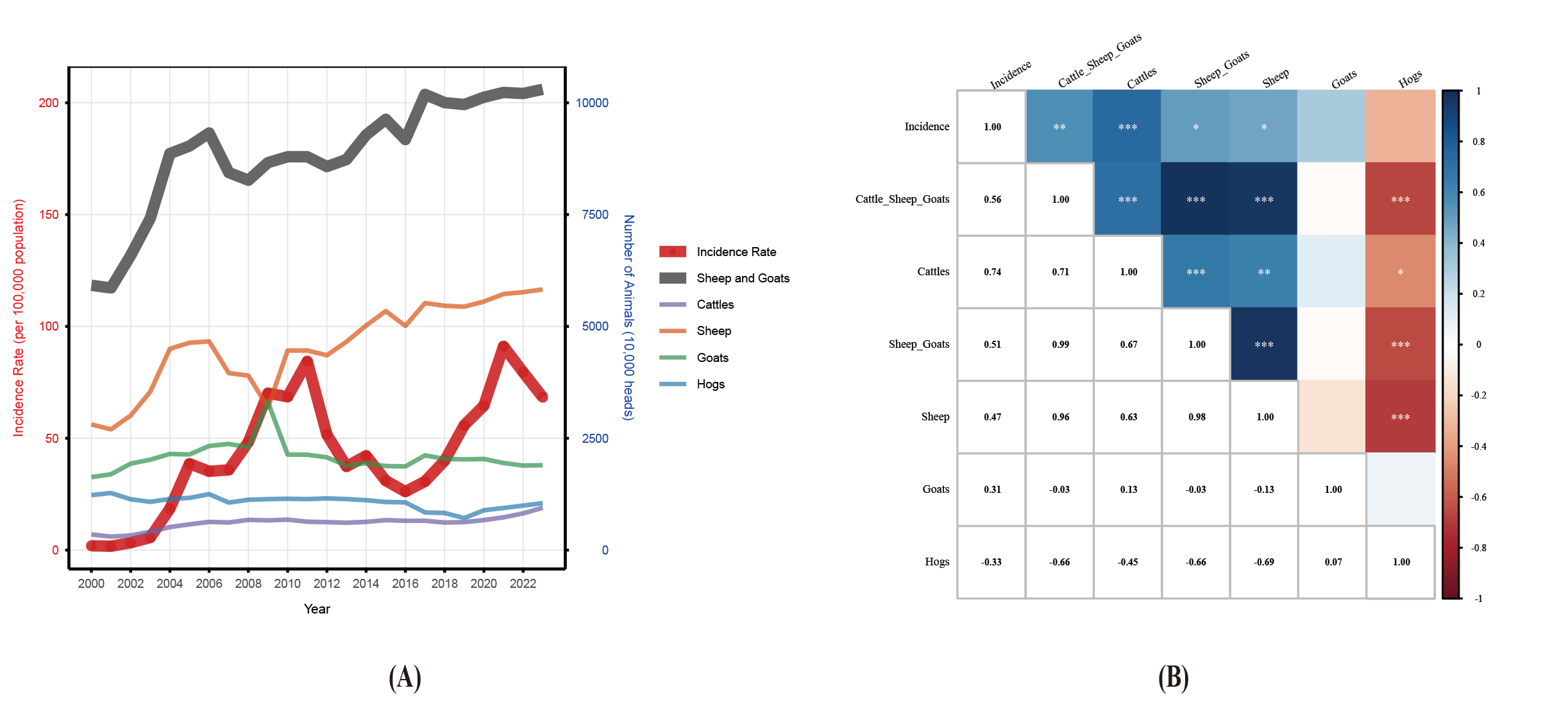

Supplement: S1 Fig — Note: (A) Temporal trends of brucellosis incidence rate (red line, per 100,000 population) and livestock population (right y-axis, in 10,000 heads) including sheep and goats (gray), cattle (purple), sheep (orange), goats (green), and hogs (blue), 2000–2023. (B) Correlation matrix showing Pearson’s correlation coefficients between brucellosis incidence and livestock populations. Blue/red colors denote positive/negative correlations, with intensity proportional to correlation magnitude. Significance levels: *p < 0.05, **p < 0.01, ***p < 0.001. (TIF) [file pntd.0014439.s001.tif]

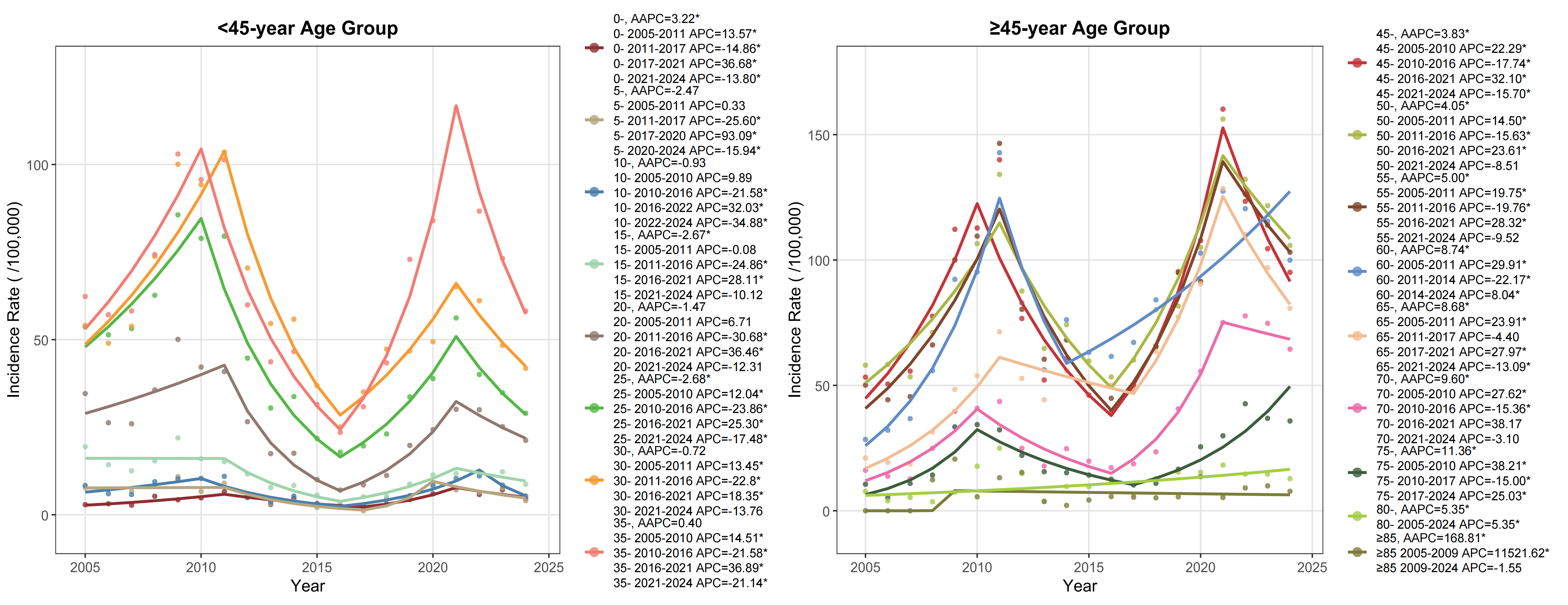

Supplement: S2 Fig — Note: APC, annual percent change. AAPC, average annual percent change. *: Statistically Significant at alpha = 0.05 (P < 0.05). (TIF) [file pntd.0014439.s002.tif]

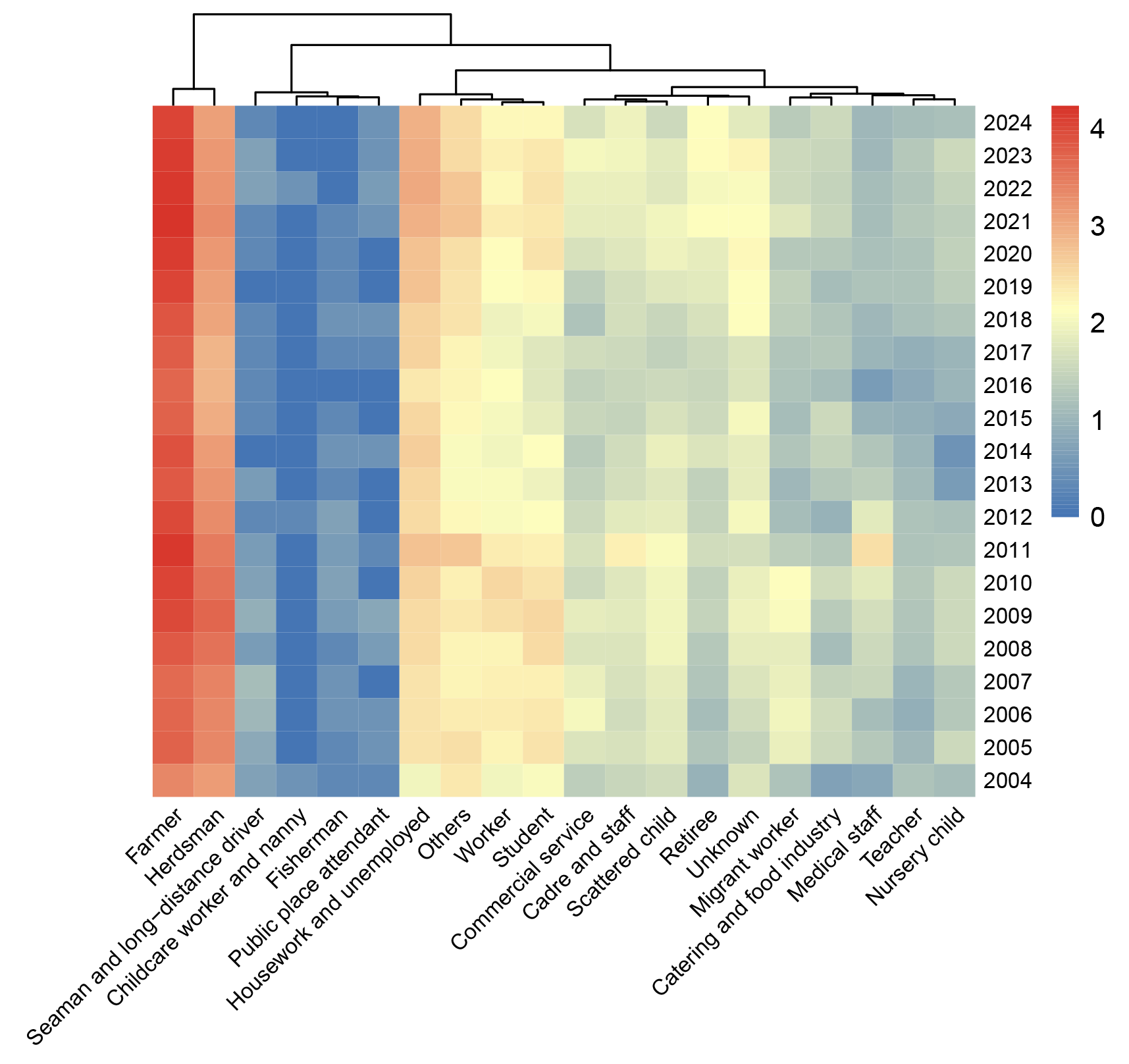

Supplement: S3 Fig — Note: Heatmap rows represent years, columns represent occupations. The color scale indicates relative case proportions (red = high, blue = low). (TIF) [file pntd.0014439.s003.tif]
